# Perceived Unmet Needs in Patients Living With Advanced Bladder Cancer and Their Caregivers: Infodemiology Study Using Data From Social Media in the United States

**DOI:** 10.2196/37518

**Published:** 2022-09-20

**Authors:** Simon Renner, Paul Loussikian, Pierre Foulquié, Benoit Arnould, Alexia Marrel, Valentin Barbier, Adel Mebarki, Stéphane Schück, Murtuza Bharmal

**Affiliations:** 1 Kap Code Paris France; 2 Icon Lyon France; 3 EMD Serono Billerica, MA United States

**Keywords:** real-world evidence, unmet needs, quality of life, social media, bladder cancer, caregivers

## Abstract

**Background:**

Locally advanced or metastatic bladder cancer (BC), which is generally termed advanced BC (aBC), has a very poor prognosis, and in addition to its physical symptoms, it is associated with emotional and social challenges. However, few studies have assessed the unmet needs and burden of aBC from patient and caregiver perspectives. Infodemiology, that is, epidemiology based on internet health-related content, can help obtain more insights on patients’ and caregivers’ experiences with aBC.

**Objective:**

The study aimed to identify the main discussion themes and the unmet needs of patients with aBC and their caregivers through a mixed methods analysis of social media posts.

**Methods:**

Social media posts were collected between January 2015 and April 2021 from US geolocalized sites using specific keywords for aBC. Automatic natural language processing (regular expressions and machine learning) methods were used to filter out irrelevant content and identify verbatim posts from patients and caregivers. The verbatim posts were analyzed to identify main discussion themes using biterm topic modeling. Difficulties or unmet needs were further explored using qualitative research methods by 2 independent annotators until saturation of concepts.

**Results:**

A total of 688 posts from 262 patients and 1214 posts from 679 caregivers discussing aBC were identified. Analysis of 340 randomly selected patient posts and 423 randomly selected caregiver posts uncovered 33 unique unmet need categories among patients and 36 among caregivers. The main unmet patient needs were related to challenges regarding adverse events (AEs; 28/95, 29%) and the psychological impact of aBC (20/95, 21%). Other patient unmet needs identified were prognosis or diagnosis errors (9/95, 9%) and the need for better management of aBC symptoms (9/95, 9%). The main unmet caregiver needs were related to the psychological impacts of aBC (46/177, 26.0%), the need for support groups and to share experiences between peers (28/177, 15.8%), and the fear and management of patient AEs (22/177, 12.4%).

**Conclusions:**

The combination of manual and automatic methods allowed the extraction and analysis of several hundreds of social media posts from patients with aBC and their caregivers. The results highlighted the emotional burden of cancer for both patients and caregivers. Additional studies on patients with aBC and their caregivers are required to quantitatively explore the impact of this disease on quality of life.

## Introduction

In 2021, approximately 84,000 people were expected to be diagnosed with bladder cancer (BC), making it the sixth most common cancer in the United States [1]. Locally advanced and metastatic stages are the aggressive stages of BC (generally termed advanced BC [aBC]) and have a poor prognosis, with the 5-year survival rate for stage IV BC estimated to be 6.4% [2]. More than 90% of BC cases involve individuals aged over 55 years, and 75% involve men [3,4].

People with BC often experience physical symptoms, including bleeding, pain, dysuria, and urinary obstruction, and they also report emotional and social challenges [5]. Patients have reported several unmet needs related to their quality of care that may vary according to disease stage and level of patient fragility, but nevertheless, there is significantly diminished physical, mental, and social quality of life (QOL) after diagnosis. A study by De Nunzio et al found that patients complained about a lack of therapeutic options for the stage of their disease [6]. Moreover, some current treatments may significantly affect QOL, specifically cystectomy, which may negatively impact mental QOL [7].

Caregivers are often responsible for managing the care of patients with BC; however, their perspective is underrepresented in the current literature. Although few studies have assessed QOL from the caregiver perspective, some recent evidence shows that caregiver QOL declines with disease stage [8]; however, more information is needed regarding their unmet needs.

Previous research on patients with BC relied on traditional research methods, such as systematic reviews [9] and questionnaires [10]. However, evidence-based practice in public health has shown some time-related challenges due to the delays that occur between data collection, publication, and the implementation of the findings. Furthermore, public health must operate on a wide scale, addressing the needs of substantial populations. This implies critical operational issues, variability, and complexity, as well as resource requirements and sustainability considerations, leading to several drawbacks, including high cost and time constraints [11]. These challenges could lead to a need for new approaches to help circumvent some obstacles of traditional methods.

Recently, social media has become increasingly compelling for obtaining valuable data concerning patients for infodemiological studies. In the early 2000s, Gunther Eysenbach first described infodemiology as a science research tool that searches the internet for health-related content posted by internet users [12]. One of the benefits of infodemiology is that it collects and analyzes high volumes of data in a time-efficient manner, in contrast to traditional methods, such as registries, questionnaires, and surveys. Thus, using technological advances instead may offer additional insights and shorten the time-consuming processes of analysis [13].

In recent years, patients with cancer have generally been increasing their use of social media networks to obtain information and support for health-related purposes [14]. Indeed, social media and online forums connect patients and caregivers to a broader patient and caregiver community with similar experiences. Within these communities, patients and caregivers seek and share support, information, advice [15], and self-care [16]. While patients with BC use the internet less often than patients with other cancers, their caregivers are active internet users [17]. Nevertheless, social media has become a novel and efficient resource for obtaining retrospective data to explore patient and caregiver perspectives about their cancer throughout the journey [15]. Previous research based on social media provided insights into patients’ and caregivers’ experiences with cancer [18] and patients’ unmet needs in regard to information and emotional support [19].

This retrospective mixed methods study aimed to identify the main discussion themes and the unmet needs that patients with aBC and their caregivers describe in their social media posts.

## Methods

### Study Design and Population

This noninterventional, retrospective, real-world, mixed methods study included data retrieved from social media posts written by patients with aBC and their caregivers. Publicly available US geolocated messages in English that were posted between January 1, 2015, and March 4, 2021, were considered. Publicly available data from social media sites (eg, Twitter) and forums (eg, patient association forums) were included. Posts on Facebook and Instagram were excluded, as most posts on these sites are private.

### Data Extraction

Data (verbatim social media posts) were identified and extracted, and irrelevant material was eliminated.

All public posts available on the web containing one of the relevant keywords were identified using the Brandwatch extractor [20]. This tool is based on queries that include selected keywords evocative of the subject of interest. Using the query, the Brandwatch extractor searches through available public data sources and identifies keywords within posts matching the ones in the query. Then, the posts, including the identified keywords, are downloaded along with their associated metadata, such as author or publication date, constituting a data set.

In this study, we constructed an extraction query (available in Multimedia Appendix 1) with keywords related to BC. The resulting data set then underwent further cleaning to exclusively obtain testimonies of patients and caregivers related to aBC. First, posts from irrelevant sources, such as potential advertising sites or forums related to pets and animals, were removed using regular expression rules. Next, a machine learning algorithm, the Extreme Gradient Boosting classifier [21], identified patient and caregiver experiences. This algorithm was previously trained on a social media data set constructed with diverse pathologies and sources of data. Predictions were formulated according to 3 variables (lexical field, syntax, and semantic style). Recorded performances in the context of training were evaluated at 78% sensitivity (ie, the proportion of identified true positives) and 69% positive predictive value (ie, the proportion of true positives among detected positives). In this study, posts pertaining to neither patients nor caregivers were filtered out. Then, a manual review was performed to ensure that the remaining posts were related to patient or caregiver experiences, thus excluding false positives. We identified posts about aBC using keywords evocating advanced levels of disease (eg, metastatic, stage IV, and advanced; see Multimedia Appendix 1 for all keywords) within the 5 words next to “bladder cancer” or “urothelial carcinoma.” The remaining data set constituted verbatim posts of patients and caregivers related to aBC. Since usernames are associated with messages, all messages from usernames containing aBC were kept in the data set even if there was no specific mention of aBC. The resulting posts were separated into one data set for patients and another for caregivers.

### Ethical Considerations

The data used in this study were obtained from sources where posts were publicly available. No private groups or web pages were accessed to gather data. When communicating or expressing themselves on the platforms included in our study, users had already consented to their data being used for other purposes.

### Data Analysis

#### Demographics

Patient or caregiver age and sex were determined by manual review, where possible (ie, when explicitly mentioned, as presented below).

[…] my dad died in August aged 68 from stage 4 bladder cancer

Otherwise, age and sex data were coded as “undetermined.”

#### Analysis of Experiences

To identify the main themes of discussion, an unsupervised automated algorithm was used to cluster posts according to their main topic. All the data were used for this analysis. To further identify difficulties and unmet needs, the annotators performed a manual qualitative analysis using a method of saturation on a random sample of the data that is described in detail below.

#### Main Themes of Discussion

To identify the main discussion themes and explore all available data about aBC, verbatim patient and caregiver posts were analyzed using a natural language processing and text mining approach called biterm topic modeling (BTM) [22,23]. BTM is a clustering method that groups similar texts according to the topics they contain. We opted to use this method in this study because of its demonstrated better performances on small-size documents [20,22]. The modeling considers posts as distributions of topics, which are themselves probability distributions over all words in the corpus. The presence of topics in posts is then used as clustering criteria. Simultaneously, for each topic, words are ordered according to their probability in this topic. The top co-occurring words can be used to label the topic through human interpretation. BTM then helps in understanding the topics of discussions of patients with aBC and their caregivers by providing a categorization of posts according to common discussed topics, described by co-occurring terms.

#### Expressed Difficulties and Unmet Needs

**Figure 1:**
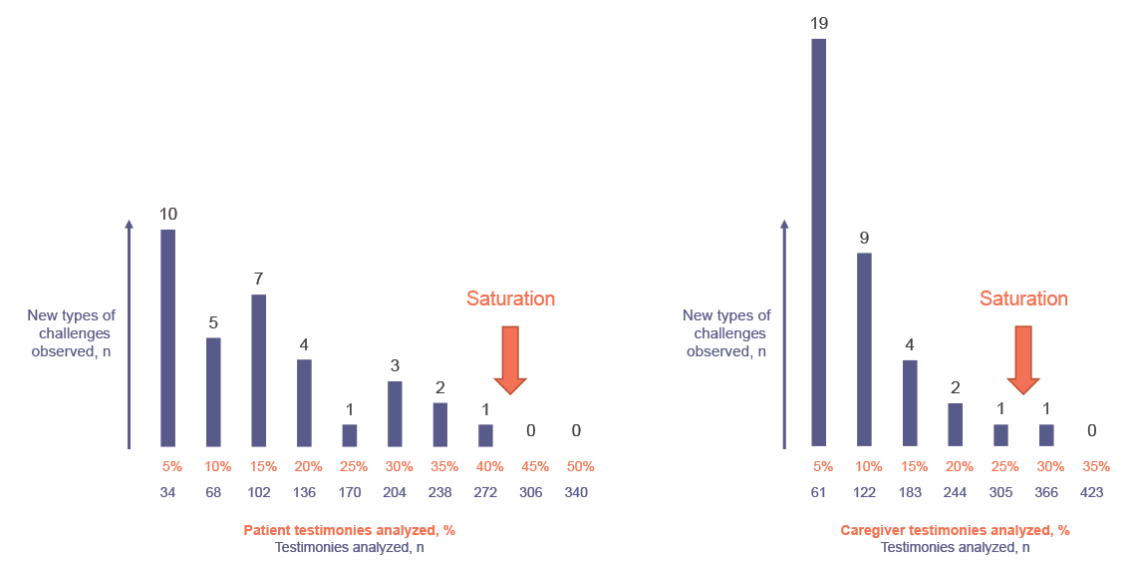
Saturation methodology for patients’ and caregivers’ posts.

To identify patients’ and caregivers’ unmet needs and categorize them, 2 independent evaluators (SR and PL) used qualitative analysis. Given the diversity of unmet needs, data saturation was used to obtain a representative sample of expressed difficulties/unmet needs. From all available posts, repeated random samples, empirically set at 5% of the total size each, were qualitatively analyzed each time until saturation was achieved. Saturation was considered achieved when 2 consecutive samples no longer yielded more than 1 new identified unmet need category (Figure 1). Two additional batches of 5% each were analyzed after saturation was first reached for further validation of our findings. As guidelines for determining saturation related to social media content are lacking, we used this novel previously described saturation approach in the qualitative analysis phase [24-26]. Difficulties were coded into distinct unmet need categories to ensure standardized data labeling and coded into whether the difficulty was related to an unmet need for the patient, caregiver, or both. For example, the following message was related to a caregiver’s unmet need:

I got some dreadful news today. My 42-year-old daughter has stage 3 bladder cancer. It has me terrified.

## Results

### Description of the Population and Posts

**Figure 2:**
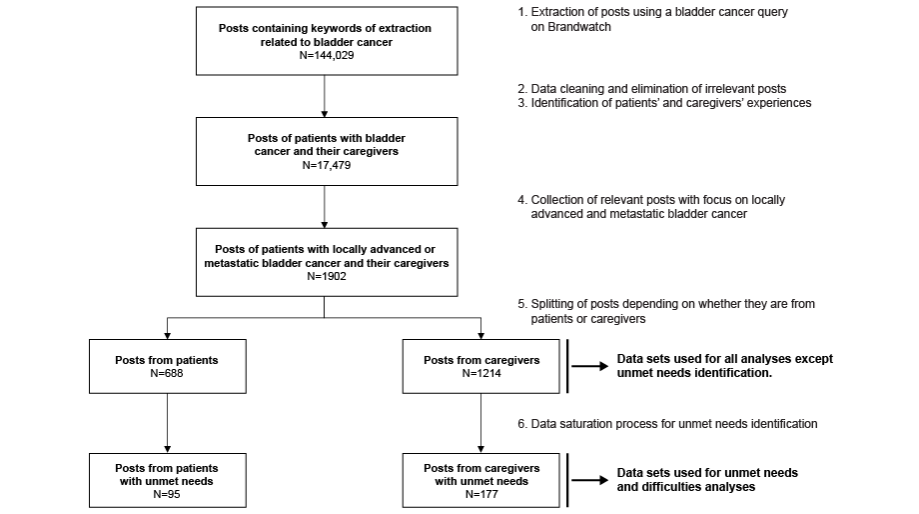
Extraction process.

The extraction yielded a total of 144,029 posts related to BC written by 68,079 users. Overall, after the cleaning step, 1214 posts from 679 caregivers and 688 posts from 262 patients were included in the study, with a median of 1.79 posts per caregiver and 2.63 posts per patient (Figure 2). The posts were retrieved from 72 caregiver discussion sources and 32 patient sources. Among them were social networks (eg, Twitter), general discussion websites (eg, Reddit), and health-related (eg, inspire website) and disease-specific (eg, bladdercancersupport website) forums. The majority of patient (139/262, 53.1%) and caregiver posts (333/679, 49.0%) came from Twitter (Table 1). Multimedia Appendix 2 contains the complete list of sources of both data sets.

Sex was mentioned for 42.4% (111/262) of patients and 15.8% (107/679) of caregivers. Age was available for 16.0% (42/262) of patients and only 3.3% (22/679) of caregivers. Among patients, 21.8% (57/262) were male; in contrast, among caregivers, only 2.8% (19/679) were male (Table 1).

**Table 1:** Patient and caregiver characteristics.

Characteristics			Patients^a^ (N=262)	Caregivers^b^ (N=679)
**Social media users, n (%)**				
	Twitter	139 (53.1)		333 (49.0)
	Reddit	16 (6.1)		95 (14.0)
	Inspire	31 (11.8)		27 (4.0)
	Bladdercancersupport	26 (9.9)		34 (5.0)
	Others	50 (19.1)		190 (28.0)
**Users’ sex, n (%)**				
	Female	54 (20.6)		88 (13.0)
	Male	57 (21.8)		19 (2.8)
	Undetermined	151 (57.6)		572 (84.2)
**Users’ age, n (%)**				
	<40 years	9 (3.4)		14 (2.1)
	40-59 years	14 (5.3)		2 (0.3)
	≥60 years	19 (7.3)		6 (0.9)
	Undetermined	220 (84.0)		657 (96.8)

^a^There were 688 posts and 32 sources.

^b^There were 1214 posts and 72 sources.

### Themes of Discussion

Using BTM separately on the whole patient and caregiver data set, posts were clustered according to their topics of discussion. Each topic was labeled and illustrated with a representative title.

The most frequent discussion themes in patient posts (Table 2) were specific to the diagnosis and different treatment possibilities, including traditional or alternative treatments, in 35.8% (246/688) of posts. Patients also exchanged messages of hope or support and shared experiences (113/688, 16.4%), while the health care pathway was addressed in 15.1% (104/688) of patient posts and included comments about patient management, screening or diagnosis methods, health care teams, etc. Symptoms and clinical signs of aBC were the dominant discussion topic in 8.4% (58/688) of patient posts. Issues related to QOL were expressed in 4.9% (34/688) of posts.

Caregivers provided messages about support and hope most frequently, accounting for 22.5% (273/1214) of posts, highlighting the sense of community that social networks might offer (Table 3). The second most frequently mentioned theme revolved around the complications of aBC (19.0% [231/1214] of posts). The third most frequent theme focused on diagnostic methods and medical procedures (18.2% [221/1214] of posts). Messages requesting scientific information about treatments were identified in 9.3% (113/1214) of posts, and themes concerning the end-of-life stage occurred in 7.6% (92/1214) of posts. Financial aspects, particularly social coverage or insurance, appeared least frequently (5.3% [64/1214] of posts).

Messages where the topic of discussion was too specific for it to constitute a main theme were pooled into the category of “other topics” (18.1% [220/1214] of caregiver messages and 19.3% [133/688] of patient messages). Some of these “other topics” included the relationship between the patient and his/her grandchildren, dementia or Alzheimer disease in relation to aBC, and the impact of COVID-19 and its repercussions.

**Table 2:** Main discussion themes in patient posts.

Themes	Patient posts (N=688), n (%)	Representative quotations
Diagnosis and different treatment possibilities, including traditional or alternative treatments	246 (35.8%)	“In 2014, I was diagnosed with metastatic bladder cancer [...]”
Exchange of messages of hope or support and sharing of patient experiences	113 (16.4%)	“My name is X. I currently have stage 4 bladder cancer and am also a retired physician. If you have any questions after your cystoscopy tomorrow let me know.”
Discussions around the health care pathway	104 (15.1%)	“Discussed chemo after the surgery with MSG oncologist Dr. X (a world Rock Star Dr). Decided to have chemo locally (I am from Delaware) instead of driving back and forth to New York City every week. My Delaware oncologist and Dr. X didn't see eye to eye on treatment.”
Symptoms and clinical signs of advanced bladder cancer	58 (8.4%)	“First was bladder cancer, tumor removed from bladder and was doing fine for a year then a lump in my arm popped up, within 6 weeks I was told to go on hospice due to Stage 4 metastatic bladder cancer that spread to my back, sacrum, hip, had it replaced, shoulder, mouth, lungs. I was free for about 2 months then it's back in my lung. Growing very slow compared to the extremely aggressive I had before.”
Focus on patient quality of life	34 (4.9%)	“[…] Occasionally, I leak from my stoma, but only when my pouch is too full. This mostly occurs at night when I don't wake up on my own to cath. (I'm past the point of setting my alarm.) I sleep with a rubber bed protector under my bottom sheet that I bought at Target in the baby section. This only happens about once every 2 weeks. I wish you well with your decision. It's a tough one, but hopefully, as you research more the diversion that fits your needs will become apparent. Good luck!!”

**Table 3:** Main discussion themes in caregiver posts.

Themes	Caregiver posts (N=1214), n (%)	Representative quotations
Sharing experiences and messages of hope and support	273 (22.5%)	“My father was recently diagnosed with stage 4 small cell bladder cancer. At first it was considered unknown primary. Can anyone please shed some light on this topic? Perhaps survivor stories. Praying and trying to stay positive.”
Complications around advanced bladder cancer	231 (19.0%)	“Hi, about 6 months ago my 88-year-old father was diagnosed with a high grade invasive bladder cancer. Unfortunately his general health also took a turn for the worse. Over the last 6 months he has been in and out of hospital with recurring chest infections. [...].”
Focus on diagnosis and medical procedures	221 (18.2%)	“My husband was diagnosed with stage 4 bladder cancer in February. In April they removed the bladder, prostate, and part of urethra, and created a neo-bladder from intestinal tissue. It worked well for about a month, then developed scar tissue and stopped working for the most part. So, since May, he has had nephrostomy tubes directly out the back from the kidneys into drainage bags.”
Scientific information on drug treatments	113 (9.3%)	“[…]Can someone tell me the most common side effects that may occur in a 72-year-old male patient given MVAC? The 5-year survival rate is 15%, but has that improved any with all of the recent clinical trials? I just want to get some advice on what to expect as I will not be with my father for a full year as I finish my studies.”
Accompanying the patient in the terminal phase and until death	92 (7.6%)	“The only regret I have was not spending everyday with my father, but at the time I didn't know a lot about cancer and thought he'd beat it.”
Discussions around social coverage, insurance, and financial aspects around patient care	64 (5.3%)	“[...] I asked how much his morphine prescription was costing. It was as much as our rent at that point, and it only increased as time went on. That was just for the painkiller, not for anything else. [...]”

### Unmet Needs

Unmet needs were identified through manual coding of a random sample from each of the patients’ and caregivers’ data sets in order to identify the most frequently expressed unmet needs.

### Patients

Among the 340 patient posts analyzed, 95 mentioned at least one difficulty among the 33 unique categories of unmet needs identified.

**Figure 3:**
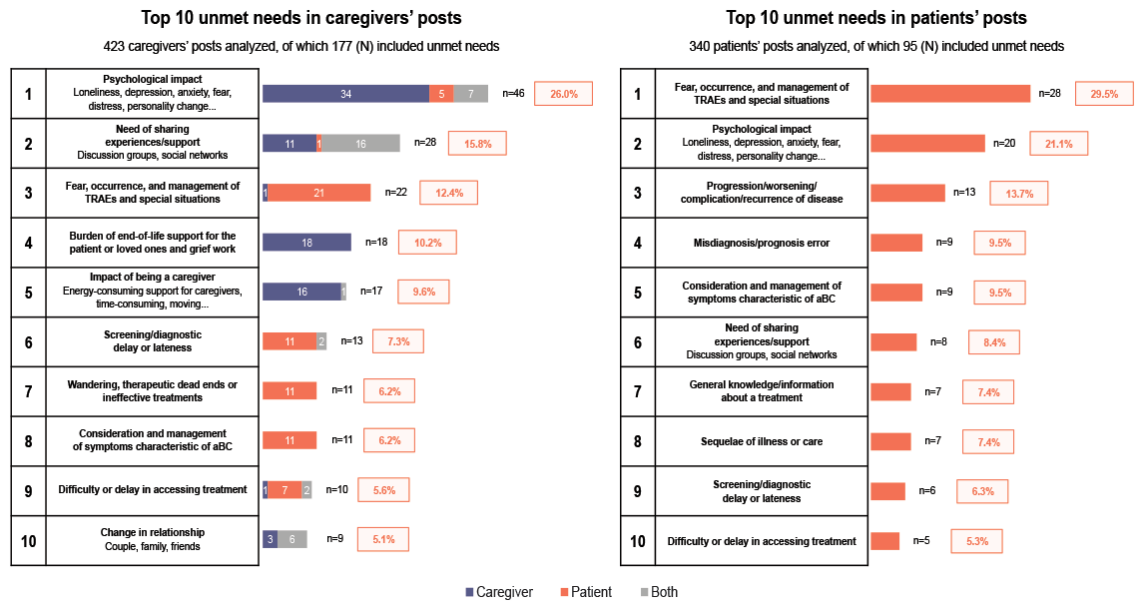
Distribution of unmet needs expressed in either patient or caregiver posts. aBC: advanced bladder cancer; TRAE: treatment-related adverse event.

Challenges concerning treatment-related adverse events (AEs) and special situations related to treatments were found in 29% (28/95) of patient posts (Figure 3). These AEs and special situations were associated with aBC treatments, including surgical procedures, as illustrated in the following message:

I had a urostomy done about 4 weeks ago, for aggressive/muscle invasive bladder cancer. My only real problem at the moment concerns night bag system kinks/leaks. During the day, I use either a one piece pouch or a one piece pouch with a leg bag (all Hollister). These options have been working great, with very few surprises/leaks. However, 3 times in the last 1.5 weeks, I have had a leak/kink/failure in my night bag system […]

Other patients discussed AEs and special situations by providing information when describing their care journey and their treatments. They share these details to provide other patients with useful knowledge and to seek advice for themselves, as reported in the following post:

I had 2 bags of Cisplatin once a week for 19 weeks. I did get neuropathy in my hands, which I still mildly have. I had cervical, ovarian, and bladder cancer. Stage 3.

A psychological impact, with feelings of loneliness, depression/discouragement, anxiety/stress, fear, or personality change, was reported in 21% (20/95) of patient posts. Difficulties with planning ahead for the future and a fear of the unknown were described in the following message:

I’m newly diagnosed with advanced bladder cancer. I'm scared about this and what's to come. Most likely, it will be chemo and removal of the bladder. Any support and input from people in the same situation will be most appreciated.

An increase in psychological distress was observed throughout the patient’s journey, starting from the diagnosis and extending to the posttreatment stage, as illustrated in the following post:

I had cervical, ovarian, and bladder cancer the 1st time. Intestinal cancer the 2nd and now 3rd time. […] So like I said, I'm a bad example. But I did have MAJOR anxiety right after I was cured the 1st time.

Other major unmet needs and challenges identified in the messages included progression, worsening, complication, or recurrence of the disease (13/95, 14%); misdiagnosis or prognostic errors (9/95, 9%); and management of symptoms characteristic of aBC, such as blood in the urine (9/95, 9%). It is noteworthy to state that no difficulties or unmet needs related to caregivers were identified during the analysis of patient posts.

### Caregivers

Among the 1214 caregiver posts analyzed, unmet needs were identified in 423 posts, 177 of which included at least one difficulty.

Psychological impact was the major difficulty caregivers described (46/177, 26.0%) (Figure 3). Similar to patients, the psychological impact was expressed throughout the patients’ health care journey, from diagnosis through treatment and management to death, as described in the following posts:

Hi there, my husband was diagnosed with stage 3 bladder cancer. […] He talked to the on call doctor. The on call Dr. said to go to the ER if there was no urine output. He seems to be urinating ok but the worry is making it worse emotionally.

I lost my dad to stage 4 bladder cancer in 2012. I was pregnant, and just trying to be the one to hold it altogether but I wasn't doing a very good job. The only thing I can say is we go through this because we have to. It's hard. It still hurts. I don't get as sad as often as I did, but when I do, its heart wrenching. Being a caretaker is exhausting and will take every ounce of energy you have emotionally and physically.

Horrifying. Just horrifying. I lost my husband last March from bladder cancer. Insidious disease.

Caregivers also expressed the need to share experiences and to support other caregivers (28/177, 15.8%). Some caregivers sought support groups for not only the patient but also themselves, as illustrated in the following message:

Is there a cancer support group on Twitter? My father has stage 4 bladder cancer and has metastasized and would love to get more info from other patients and survivors.

Caregivers also solicited advice from people with similar experiences, requesting information, guidance, and recommendations, as follows:

My father was diagnosed with invasive bladder cancer in February 2016. […] We are now running into several problems which have resulted in readmission into the hospital on several occasions. I would like to hear if others have encountered similar issues. […] I realize this may be a constant battle but I need any tips possible. He drinks constantly but with the new “bladder” and stoma I feel he loses more water. Tips/advice? […] Please share your journey, hardships, and advice. I really want to help him get through this and provide the best possible life and outcome for him.

Alternatively, some posts expressed the need for spiritual support with requests for prayers, for example:

So my dad has bladder cancer. Mom is taking it pretty hard. Please keep my family in your prayers if you think about it. Thank you. #cancersucks

Challenges concerning AEs and specific treatment-related situations were found in 12.4% (22/177) of caregiver posts. Posts about AEs specifically concerned the patient and mentioned one or more AEs, the potential management, and the fear either patients or caregivers feel regarding the impact of the AEs, as expressed in the following message:

My 84-year-old father was diagnosed in May 2016 with a high grade urothelial bladder cancer. […] The chemo was very harsh on my dad and set him back...dropping his blood count & platelets numerous times requiring blood transfusions & platelets.

Caregivers also expressed challenges associated with the burden of end-of-life support and the grief of losing a loved one (18/177, 10.2%), being a caregiver (17/177, 9.6%), having a late diagnosis or delayed screening (13/177, 7.3%), and aBC symptom management (11/177, 6.2%), for example:

[…] No, it doesn't get any easier. The fear, the waiting and sitting around the house is like being on death row. Cancer not only is killing the patient, it’s killing the family too.

## Discussion

### Principal Findings

To our knowledge, this is the first study to analyze data retrieved from social media posts written by patients with aBC and their caregivers to gain insights into the perspectives of both the patient and caregiver about the difficulties encountered when living with aBC. We found that a majority of caregiver concerns focused on the psychological impact of aBC, whereas patients mainly focused on managing AEs. Our findings also support recent literature suggesting that patients particularly need psychological support and information about aBC and its treatment [17,27,28]. These insights are particularly valuable as they are based on analysis of open-ended verbatim posts, which are typically not captured using traditional survey methods.

Interestingly, we found that almost twice as many caregivers as patients had posted online about aBC (1214 vs 688). Although few patients or caregivers who posted specified their age and sex, most caregivers who did were women aged between 30 and 40 years, whereas most patients who did were men aged between 50 and 60 years. These patients who were active on social media were among the younger population living with aBC. Older patients tended to post much less, possibly because of lower electronic literacy [29]. These findings are consistent with those of a recent review suggesting that caregivers actively use the internet to access information on behalf of patients [30]. Furthermore, this lower representation of people with aBC accessing information online may just be due to the fact that aBC is more prevalent in older men [3,4].

Treatment, psychological impact, and disease and symptom management were the unmet needs that patients discussed most often, which is in accordance with the few published studies highlighting the challenges faced by patients. These challenges include making long- and short-term treatment decisions [5]; the need for equipment support (eg, support using stomal appliances, catheters, and incontinence) [5]; the need for informational, intimacy, and psychological support [31-33]; and improving mental health [34]. These latter challenges are particularly important, because supporting psychological needs and improving mental health can positively impact treatment outcomes and survival-related outcomes [34].

Furthermore, our findings confirmed the negative impact that aBC has on caregiver QOL. This is consistent with results from other studies on the short-term [35,36] and long-term [37] burden on the caregivers of people with other cancer types. The main unmet need caregivers expressed in this study was a lack of support, which drove them to seek support and advice on social media. Caregivers also highlighted that the time and energy required for end-of-life logistics and support were particularly challenging. We found that social media provided caregivers with a forum where they could convey their concerns and express both their challenges in caring for someone with aBC and the challenges faced by the patient. The unmet needs and challenges highlighted in this study emphasize the importance of considering the caregiver’s role and needs, and not just the patient’s role and needs.

This information may be used in the development of personalized and holistic approaches, centering around the needs of patients and their caregivers. The data presented may help health care professionals to further grasp the impact of the disease on their patients, which in turn will enhance the management of their health care journey. Feedback on social media may be used for health monitoring, developing initiatives for patients with BC, and developing targeted awareness campaigns.

### Study Strengths and Limitations 

The strengths of this study include a mixed method approach combining natural language processing with qualitative analysis. The study analyzed data from a 6-year period and included a large sample size. Furthermore, open-ended verbatim posts were analyzed, providing more data than traditional survey methods.

However, the observational nature based on social media data has some limitations. The posts extracted were limited to publicly available messages, thus excluding Facebook and Instagram. Moreover, relevant posts may have inadvertently been excluded during the filtering process. Data from social media posts may have been limited by the selected information and perspectives that patients and caregivers chose to post, depending on their technological literacy, BC experience, and understanding of key medical aspects. This means that some key contextual information, such as disease stage or specific treatment information, may not have been captured. Additionally, our study was subject to selection bias, as patient and caregiver posts may not be representative of all patients with BC or aBC and their caregivers. Indeed, the level of social media engagement differs according to age, sex, socioprofessional level, education, and technological literacy.

The natural processing analysis may have been limited by the threshold values chosen to reduce background noise, which were set empirically in this study, similar to our previous work [36,38,39]. Lastly, the saturation method used to identify unmet needs was only applied to random data extracts as opposed to analyzing the full data set. Thus, it is possible that saturation was not met in the full data set, and some perspectives or unmet needs may have been missed. Despite these limitations, this study offers valuable findings on the unmet needs of patients living with aBC and their caregivers, based on their direct inputs.

### Future Work and Impact on Care

This study identified leverage points to improve the patient experience. Both patients and caregivers described the psychological impact that aBC has on them and the need for clear BC information and practical advice. Patients also reported the need for clearer communication between themselves and practitioners [33]. Patient-physician online interaction about BC is less developed than that for other cancers. Breast cancer has the largest online community, and online discussions have existed for many years [18]. The social media output for prostate cancer is increasing, particularly on Twitter [27]. Furthermore, a recent study rated the quality of BC content available on YouTube as moderate to poor, meaning that patients are at risk of being exposed to misinformation and potential harm [28]. This highlights the need for clear, accessible, and accurate information about BC and its treatment and management. Emotional support to counteract the psychological impact of BC is also essential. The use of social media is a method that could be adopted to help meet these needs.

As previously shown in the literature, difficulties for people with BC differ according to age, sex, and treatment [40]. However, Grov and Valeberg highlighted a similar impact on caregiver mental health and decreased QOL regardless of disease stage [41]. Future research is required to explore whether this is reflected on social media.

The results of this study may help raise awareness about patient and caregiver unmet needs with health care professionals. This could help ensure that patients receive holistic patient-centered treatment that does not focus solely on the aBC but considers the patient as a whole. Furthermore, it could help to improve available information, communication, and support for both patients and caregivers.

Our innovative data analysis method combined the BTM method, a well-accepted natural language processing technique for analyzing social media posts [39,42,43], with qualitative analysis of a random data sample coupled with saturation. These 2 complementary methods could be used to explore unmet needs or perspectives expressed on social media about other diseases.

### Conclusions

Social media and online forums are innovative and efficient resources for obtaining data on patient and caregiver perspectives about aBC, which may be difficult to assess through traditional research methods. These online forums complement real-world evidence for unmet needs in specific populations.

People living with aBC mostly expressed unmet needs concerning treatment, psychological impact, or disease and symptom management, whereas caregivers expressed the emotional burden of caring, especially during end-of-life stages, as well as the need for support. These data may help raise awareness about these unmet needs, which may otherwise have remained unknown if patients and caregivers had not posted these perceptions on social media, among health care professionals and clinicians.

## Appendix

Multimedia Appendix 1 Query and keywords used.

Multimedia Appendix 2 Sources of patient and caregiver posts.

## Abbreviations

aBC: advanced bladder cancer
AE: adverse event
BC: bladder cancer
BTM: biterm topic modeling
QOL: quality of life
