# Supplementary material for: Perceived Unmet Needs in Patients Living With Advanced Bladder Cancer and Their Caregivers: Infodemiology Study Using Data From Social Media in the United States
Source: JMIR Cancer. 2022 Sep 20;8(3):e37518. doi: 10.2196/37518 (PMC9533198; doi:10.2196/37518)
Supplement: Multimedia Appendix 1 [file cancer_v8i3e37518_app1.docx]

## Appendix 1

### Query used for extraction on Brandwatch

((cancer OR carcinoma OR tumor) NEAR/3 (bladder OR urothelial OR urotelial)) OR bavencio OR avelumab OR (BC NEAR/6 (bladder OR uro*)).

The operator “NEAR/X” was used to combinate 2 words or expression within the X adjacent words. The operator “*” was used to identify mentions with the root word “uro” (eg, urological, urothelial).

### Keywords used to select posts regarding aBC

Advanced

Invasive

Locally advanced

Lymph node

M=1

M1

Metastasis

Metastasized

Metastatic

MIBC

Muscle invasive

Muscle invasive cancer

Muscle invasive metastatic

Muscle invasive non metastatic

Muscle invasive non-metastatic

Muscle-invasive

Muscle-invasive cancer

Muscle-invasive non-metastatic

N=1

N=2

N=3

N1

N2

N3

Stage 3

Stage 3a

Stage 3b

Stage 4

Stage 4a

Stage 4b

Stage III

Stage IIIa

Stage IIIb

Stage IV

Stage IVa

Stage IVb

T=3

T=3a

T=3b

T=4

T=4a

T=4b

T3

T3a

T3b

T4

T4a

T4b

Terminal
