# Supplementary material for: Perceived Unmet Needs in Patients Living With Advanced Bladder Cancer and Their Caregivers: Infodemiology Study Using Data From Social Media in the United States
Source: JMIR Cancer. 2022 Sep 20;8(3):e37518. doi: 10.2196/37518 (PMC9533198; doi:10.2196/37518)
Supplement: Multimedia Appendix 2 [file cancer_v8i3e37518_app2.docx]

## Appendix 2

### All the sources of posts for patients with aBC

| **Source** | **No. of posts, n (%)** | **No. of users, n (%)** |
| --- | --- | --- |
| Twitter.com | 364 (52.9) | 97 (37.0) |
| Inspire.com | 84 (12.2) | 43 (16.4) |
| Bladdercancersupport.org | 67 (9.7) | 58 (22.1) |
| Reddit.com | 39 (5.7) | 16 (6.1) |
| Cafemom.com | 20 (2.9) | 1 (0.4) |
| Ic-network.com | 20 (2.9) | 1 (0.4) |
| Newagtalk.com | 16 (2.3) | 1 (0.4) |
| Cancercompass.com | 10 (1.4) | 5 (1.9) |
| Breast cancer | 9 (1.3) | 2 (0.8) |
| Cancer.org | 9 (1.3) | 8 (3.0) |
| Patient.info | 9 (1.3) | 4 (1.5) |
| Delphiforums.com | 5 (0.7) | 2 (0.8) |
| Avvo.com | 4 (0.6) | 1 (0.4) |
| Babycenter.com | 4 (0.6) | 2 (0.8) |
| Mdjunction.com | 3 (0.4) | 1 (0.4) |
| Navigatingcancer.com | 3 (0.4) | 2 (0.8) |
| Studentdoctor.net | 3 (0.4) | 1 (0.4) |
| Dcurbanmom.com | 2 (0.3) | 2 (0.8) |
| Oncotherapynetwork.com | 2 (0.3) | 1 (0.4) |
| Supportgroups.com | 2 (0.3) | 2 (0.8) |
| Thelion.com | 2 (0.3) | 1 (0.4) |
| Alzconnected.org | 1 (0.2) | 1 (0.4) |
| Cancernetwork.com | 1 (0.2) | 1 (0.4) |
| Drugs.com | 1 (0.2) | 1 (0.4) |
| Ehealthforum.com | 1 (0.2) | 1 (0.4) |
| Healthcaremagic.com | 1 (0.2) | 1 (0.4) |
| Hubpages.com | 1 (0.2) | 1 (0.4) |
| Livestrong.com | 1 (0.2) | 1 (0.4) |
| Medhelp.org | 1 (0.2) | 1 (0.4) |
| Psychcentral.com | 1 (0.2) | 1 (0.4) |
| Regulations.gov | 1 (0.2) | 1 (0.4) |
| Twoplustwo.com | 1 (0.2) | 1 (0.4) |

### All the sources of caregiver posts (caregivers of patients with aBC)

| **Source** | **No. of posts, n (%)** | **No. of users, n (%)** |
| --- | --- | --- |
| Twitter.com | 430 (48.0) | 310 (45.7) |
| Reddit.com | 126 (14.1) | 92 (13.6) |
| Bladdercancersupport.org | 53 (5.9) | 51 (7.5) |
| Babycenter.com | 41 (4.6) | 30 (4.4) |
| Inspire.com | 32 (3.6) | 24 (3.5) |
| Cancercompass.com | 20 (2.2) | 13 (1.9) |
| Cancer.org | 18 (2.0) | 15 (2.2) |
| Breastcancer.com | 14 (1.6) | 7 (1.0) |
| City-data.com | 13 (1.4) | 10 (1.5) |
| Navigatingcancer.com | 11 (1.2) | 10 (1.5) |
| Talkingaboutmenshealth.com | 9 (1.0) | 1 (0.2) |
| Mumsnet.com | 7 (0.8) | 6 (0.9) |
| Proboards.com | 7 (0.8) | 5 (0.7) |
| Agingcare.com | 6 (0.7) | 4 (0.6) |
| Change.org | 6 (0.7) | 6 (0.9) |
| Healingwell.com | 6 (0.7) | 3 (0.4) |
| Cancer-forums.net | 5 (0.6) | 5 (0.7) |
| Cancerforums.net | 5 (0.6) | 4 (0.6) |
| Cancergrace.org | 5 (0.6) | 4 (0.6) |
| Democraticunderground.com | 5 (0.6) | 3 (0.4) |
| Straightdope.com | 5 (0.6) | 1 (0.2) |
| Thebump.com | 5 (0.6) | 3 (0.4) |
| Google.com | 4 (0.4) | 2 (0.3) |
| Hogville.net | 4 (0.4) | 2 (0.3) |
| Youbemom.com | 4 (0.4) | 4 (0.6) |
| Dailykos.com | 3 (0.3) | 2 (0.3) |
| Dcurbanmom.com | 3 (0.3) | 3 (0.4) |
| Delphiforums.com | 3 (0.3) | 2 (0.3) |
| Diabetesdaily.com | 3 (0.3) | 1 (0.2) |
| Legacy.com | 3 (0.3) | 3 (0.4) |
| Medhelp.org | 3 (0.3) | 2 (0.3) |
| Melanoma.org | 3 (0.3) | 2 (0.3) |
| Sigforum.com | 3 (0.3) | 1 (0.2) |
| Weightwatchers.com | 3 (0.3) | 2 (0.3) |
| Zetaboards.com | 3 (0.3) | 2 (0.3) |
| Alzconnected.org | 2 (0.2) | 1 (0.2) |
| Colonclub.com | 2 (0.2) | 1 (0.2) |
| Facmedicine.com | 2 (0.2) | 2 (0.3) |
| Fark.com | 2 (0.2) | 2 (0.3) |
| Fool.com | 2 (0.2) | 1 (0.2) |
| Freerepublic.com | 2 (0.2) | 2 (0.3) |
| Her2support.org | 2 (0.2) | 1 (0.2) |
| Ihatedialysis.com | 2 (0.2) | 2 (0.3) |
| Justanswer.com | 2 (0.2) | 2 (0.3) |
| Mdjunction.com | 2 (0.2) | 1 (0.2) |
| Psychcentral.com | 2 (0.2) | 1 (0.2) |
| Resetera.com | 2 (0.2) | 1 (0.2) |
| Rivals.com | 2 (0.2) | 2 (0.3) |
| Tc-cancer.com | 2 (0.2) | 1 (0.2) |
| Wgmd.com | 2 (0.2) | 1 (0.2) |
| Yellowbullet.com | 2 (0.2) | 1 (0.2) |
| Yuku.com | 2 (0.2) | 2 (0.3) |
| Zyngaplayerforums.com | 2 (0.2) | 1 (0.2) |
| 4channel.org | 1 (0.1) | 1 (0.2) |
| Anxiety-central.com | 1 (0.1) | 1 (0.2) |
| Anxietyzone.com | 1 (0.1) | 1 (0.2) |
| Biology-forums.com | 1 (0.1) | 1 (0.2) |
| Boards.net | 1 (0.1) | 1 (0.2) |
| Digitalspy.com | 1 (0.1) | 1 (0.2) |
| Godlikeproductions.com | 1 (0.1) | 1 (0.2) |
| Healthboards.com | 1 (0.1) | 1 (0.2) |
| Healthcaremagic.com | 1 (0.1) | 1 (0.2) |
| Houzz.com | 1 (0.1) | 1 (0.2) |
| Medicalnewstoday.com | 1 (0.1) | 1 (0.2) |
| Pprune.org | 1 (0.1) | 1 (0.2) |
| Qvc.com | 1 (0.1) | 1 (0.2) |
| Rimfirecentral.com | 1 (0.1) | 1 (0.2) |
| Topix.net | 1 (0.1) | 1 (0.2) |
| Trailerlife.com | 1 (0.1) | 1 (0.2) |
| Trapperman.com | 1 (0.1) | 1 (0.2) |
| Welltrainedmind.com | 1 (0.1) | 1 (0.2) |
| Yahoo.com | 1 (0.1) | 1 (0.2) |
